# Oxidative Stress at Birth Is Associated with the Concentration of Iron and Copper in Maternal Serum

**DOI:** 10.3390/nu13051491

**Published:** 2021-04-28

**Authors:** Karolina Rak, Karolina Łoźna, Marzena Styczyńska, Łukasz Bobak, Monika Bronkowska

**Affiliations:** 1 Department of Human Nutrition, Faculty of Biotechnology and Food Science, Wrocław University of Environmental and Life Sciences, 51-630 Wrocław, Poland; karolina.lozna@upwr.edu.pl (K.Ł.); marzena.styczynska@upwr.edu.pl (M.S.); monika.bronkowska@upwr.edu.pl (M.B.); 2 Department of Functional Food Products Development, Faculty of Biotechnology and Food Science, Wrocław University of Environmental and Life Sciences, 51-630 Wrocław, Poland; lukasz.bobak@upwr.edu.pl

**Keywords:** oxidative stress, 3′nitrotyrosine, nutritional status, mineral elements, pregnant women

## Abstract

Oxidative stress (OS) in the foetal and neonatal periods leads to many disorders in newborns and in later life. The nutritional status of pregnant women is considered to be one of the key factors that triggers OS. We investigated the relationship between the concentration of selected mineral elements in the blood of pregnant women and the concentration of 3′nitrotyrosine (3′NT) as a marker of OS in the umbilical cord blood of newborns. The study group consisted of 57 pregnant women and their newborn children. The concentrations of magnesium (Mg), calcium (Ca), iron (Fe), zinc (Zn) and copper (Cu) in maternal serum (MS) were measured by the flame atomic absorption/emission spectrometry (FAAS/FAES) method. The concentration of 3′NT in umbilical cord serum (UCS) of newborns was determined by the ELISA method. A positive correlation between MS Fe and UCS 3′NT in male newborns was shown (rho = 0.392, *p* = 0.053). Significantly higher UCS 3′NT was demonstrated in newborns, especially males, whose mothers were characterized by MS Fe higher than 400 μg/dL compared to those of mothers with MS Fe up to 300 μg/dL (*p* < 0.01). Moreover, a negative correlation between the MS Cu and UCS 3′NT in male newborns was observed (rho = −0.509, *p* = 0.008). Results of the study showed the need to develop strategies to optimize the nutritional status of pregnant women. Implementation of these strategies could contribute to reducing the risk of pre- and neonatal OS and its adverse health effects in the offspring.

## 1. Introduction

Oxidative stress (OS) is a state of disturbed balance between oxidation and reduction processes. There is an overproduction of reactive oxygen and nitrogen species (ROS/RNS), which causes structural changes and damage to lipid molecules, nucleic acids and proteins, resulting in modification of their biological functions or complete deactivation [1,2,3,4,5]. 

**Figure 1:**
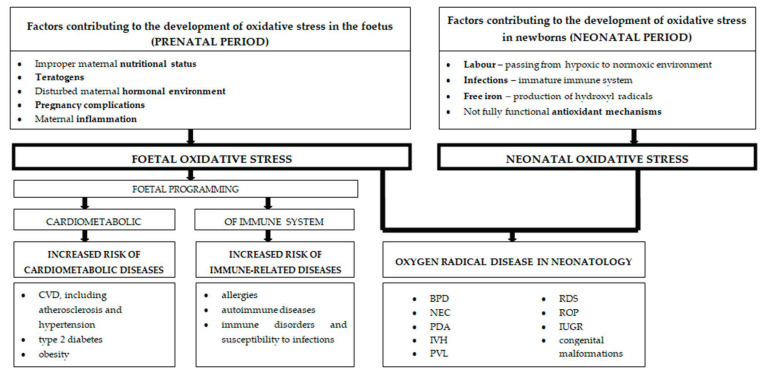
The impact of pre- and neonatal oxidative stress on the health of newborns and health predispositions in later life. CVD—cardiovascular disease; BPD—bronchopulmonary dysplasia; NEC—necrotizing enterocolitis; PDA—patent ductus arteriosus; IVH—intraventricular haemorrhage; PVL—periventricular leukomalacia; RDS—respiratory distress syndrome; ROP—retinopathy of prematurity; IUGR—intrauterine growth restriction.

OS is especially unfavourable in the pre- and neonatal period, when intensively multiplying cells and developing tissues of young organisms are particularly sensitive to the harmful effects of ROS/RNS produced under the influence of various factors [6,7,8,9,10,11,12,13]. OS in early life may result in the development of “oxygen radical disease in neonatology” [14,15,16,17,18,19,20] as well as foetal programming [6,21,22], that determines susceptibility to cardiometabolic [6,21,23,24,25,26] and immunological diseases in later life [7,27,28,29,30,31,32]. Figure 1 shows the triggers of OS in the pre- and neonatal period and its health consequences in newborns and later in life. 

During foetal life, many factors can induce OS in the foetus—(1) teratogens (e.g., chemicals, pathogenic microorganisms, drugs, alcohol and tobacco), (2) disturbed hormonal environment (resulting from maternal obesity, excessive weight gain in pregnancy, diabetes and stress), (3) complications in the course of pregnancy (including pre-eclampsia, gestational hypoxia and placental insufficiency), and (4) inflammation (resulting from infection) [6,7,8,9]. 

The improper nutritional status (insufficient, excessive or unbalanced) of pregnant women has also been demonstrated to be one of the key maternal pro-oxidative factors that triggers, by activating the hypothalamic-pituitary-adrenal (HPA) axis [27], a stressful intrauterine environment and can lead to foetal and neonatal OS [6,7,8,9]. Increased demand for nutrients during pregnancy very often results in their deficiency [33,34,35,36,37,38]. On the other hand, the use of dietary supplements by pregnant women may contribute to the excessive supply of nutrients, especially vitamins and mineral elements [39,40]. Inappropriate proportions of nutrients in the diet, especially in the Western model of nutrition, may also result in disturbance of nutritional status of pregnant women [41]. 

Mineral elements play many important biological roles in the human organism [42,43], including in the redox balance. Iron (Fe), copper (Cu) and zinc (Zn)—trace elements from the group of transition metals—are able to adopt various oxidation states by accepting or donating electrons from the outer shell. Therefore, they can act as pro- and antioxidants and their concentration in the body is strictly regulated [44,45,46]. As both insufficiency and excess of Fe, Cu and Zn have been demonstrated to cause OS, the relationship between the concentration of these mineral elements in the body and the level of OS is defined as U-shaped [47,48,49,50].

In physiological conditions, Fe is involved in the production of ROS/RNS as an antimicrobial defence mechanism [51], while Fe overload is considered one of the major pro-oxidative factors by driving the Fenton reaction and the production of highly reactive hydroxyl radicals [49]. In turn, Cu and Zn play a role in antioxidant processes as components of copper-zinc superoxide dismutase (Cu-Zn SOD) which is one of the key elements of intracellular antioxidant mechanisms [44,50]. Insufficient levels of Cu and Zn may promote OS by impairing antioxidant mechanisms [44,50,52,53]. 

Magnesium (Mg) has been demonstrated to influence the level of OS in the organism [54] and its insufficiency (hypomagnesaemia) can promote ROS/RNS production [55]. In turn, calcium (Ca) is essential for the proper course of many stages of the reproductive process, including the development of the placenta [56]—a key structure ensuring intrauterine homeostasis and buffering the influence of adverse stimuli on the developing foetus. 

Although in the literature there are presented results suggesting that both insufficiency and excess of mineral elements in pregnancy may have adverse effects for foetal development, maternal health and health of their offspring [33,57], according to our best knowledge, there are no studies that examined the relationship between status of mineral nutrition in pregnancy and the level of OS in newborns.

We therefore aimed to investigate the relationship between the concentration of mineral elements (Ca, Mg, Fe, Cu, Zn) in the blood of pregnant women and the concentration of 3′nitrotyrosine (3′NT) as an OS marker in the umbilical cord blood of newborns. We hypothesized that an insufficient or an excessive concentration of examined mineral elements in maternal serum may increase the level of OS in their newborn children. 

## 2. Materials and Methods 

### 2.1. Patients

A total of 57 full-term healthy neonates (31 females and 26 males) born in the Obstetrics and Gynaecology Ward of Provincial Specialist Hospital in Wroclaw Research and Development Centre (Poland) between April 2016 and December 2016 and their mothers were enrolled in the study. All examined women declared no alcohol and tobacco use throughout the pregnancy and had uncomplicated pregnancies terminated with caesarean section (no labour) due to obstetric (previous caesarean section), ophthalmological (advanced eye defect) or psychiatric (tokophobia) indications. Women with natural labour were not enrolled in the study. Characteristics of mothers and newborns are presented in Table 1, taking into account the data on maternal age, pre-pregnancy BMI, gestational weight gain, concentrations of selected mineral elements in the MS, gestational age of newborns, their birth weight and concentration of 3′NT in the UCS. Moreover, the frequency of insufficient, optimal and excessive concentrations of selected mineral elements in the blood serum of pregnant women in relation to the reference values for pregnant women in the 3rd trimester [58] are presented in Table 2.

### 2.2. Determination of Concentration of Mineral Elements in Maternal Serum 

Maternal blood samples were collected before planned caesarean section (no labour). Blood samples after clotting were centrifuged for 15 min at 2000 rpm. Serum samples were removed and stored at −80 °C until the analysis [59]. The concentrations of Ca, Mg, Zn, Fe and Cu in maternal serum (MS) were measured by means of flame absorption (emission for Ca) atomic spectrometry (FAAS/FAES) with the use of a spectrometer (SpectraAA 240 FS, Varian). Previously, serum samples were wet mineralized. Reaction mixtures consisting of 1 g of serum, 5 cm^3^ of 65% nitric acid (V) and 1 cm^3^ of hydrogen peroxide, after incubation overnight at room temperature, were mineralized in a closed microwave system MARS 5 over 10 min at 180 °C and 180 psi and then transferred quantitatively into volumetric flasks of 10 cm^3^ using redistilled water [60,61,62]. The concentrations of Ca and Mg were expressed in mg/dL, while the concentrations of Zn, Fe and Cu were expressed in μg/dL. The concentrations of mineral elements in MS were classified using reference values for pregnant women in the 3rd trimester (Table 2) [58]. 

### 2.3. Determination of 3′Nitrotyrosine in Cord Blood Serum of Newborns as an Oxidative Stress Marker

Umbilical cord blood samples were collected during caesarean section. Blood samples after clotting were centrifuged for 15 min at 2000 rpm. Serum samples were removed and stored at −80 °C until the analysis [59]. The concentration of 3′NT in umbilical cord serum (UCS) was measured in duplicate with enzyme-linked immunosorbent assay (General Nitrotyrosine, 3NT ELISA Kit, EIAab, Wuhan, China). The absorption was measured with an Epoch plate reader (Bio-Tek Instruments, Winooski, VT, USA). Concentrations of 3′NT were expressed as pg/mL. 

### 2.4. Statistical Analysis

Statistical analysis was performed with STATISTICA software, version 10.0. Spearman’s correlation was applied to estimate the association between continuous variables (no conditions for *t* test). The Mann-Whitney test was used to compare the rank between two groups of independent variables while the Kruskal–Wallis test was used to compare the variance between more than two groups of independent variables. For all analyses, *p* ≤ 0.05 was considered significant [63]. In the Results section, the size of subgroups (N), median values (Me) and interquartile range (IQR) are presented. 

### 2.5. Ethical Approval

The study was approved by the Bioethics Committee at the Medical University of Wroclaw, Poland (record number KB-158/2016). All pregnant women were informed about the aims of the study and gave their written informed consent. 

## 3. Results

**Figure 2:**
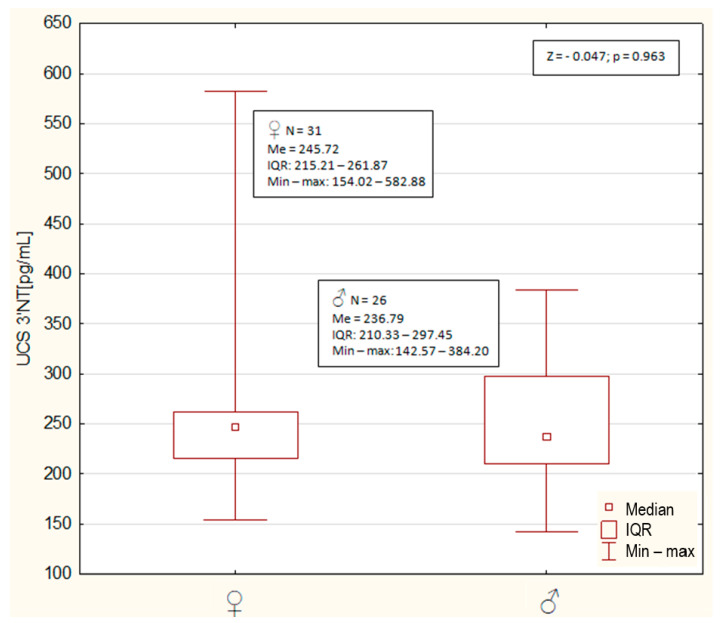
Median concentrations (Me), interquartile range (IQR) and the range (min-max) of 3′nitrotyrosine in cord blood serum (UCS 3′NT) of female (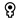) and male (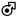) newborns. Descriptive statistics of subgroups, value of Mann–Whitney test (Z) and *p* value are presented in frames.

Figure 2 presents median concentrations of 3′NT in the UCS in newborns in relation to sex. No statistically significant difference in 3′NT in UCS was demonstrated between female and male newborns (*p* = 0.963).

Results of Spearman’s correlation between the concentrations of Mg, Ca, Zn, Fe and Cu in the MS and the concentration of 3′NT in the UCS of all newborns (regardless of sex), female newborns and male newborns are presented in Table 3. A statistically significant negative correlation was observed between the concentration of Cu in the MS and the concentration of 3′NT in the UCS, but only in male newborns (rho = −0.509, *p* = 0.008). Simultaneously, the concentration of 3′NT in the UCS of male newborns was positively correlated at the edge of statistical significance (rho = 0.392, *p* = 0.053) with the concentration of Fe in the MS. No significant relationship of examined mineral elements with OS in female newborns and all newborns was found. 

The concentration of 3′NT in the UCS in relation to the concentration of selected mineral elements in the MS (insufficient, optimal and excessive) and the results of Mann–Whitney tests between subgroups are presented in Table 4. No significant differences were demonstrated in the concentration of 3′NT in UCS between newborns whose mothers had insufficient, excessive or optimal concentrations of Mg, Fe and Cu in the MS. As all the examined pregnant women were characterized by an excessive level of Ca and the vast majority of them had insufficient levels of Zn, division into quartiles of these mineral elements was applied. The concentration of 3′NT in the UCS in relation to the concentration of Ca and Zn in the MS (Q_1_, Q_2_, Q_3_, Q_4_) and the results of Kruskal–Wallis tests between subgroups are presented in Table 5. No significant differences in 3′NT in the UCS between the analysed subgroups were observed.

**Figure 3:**
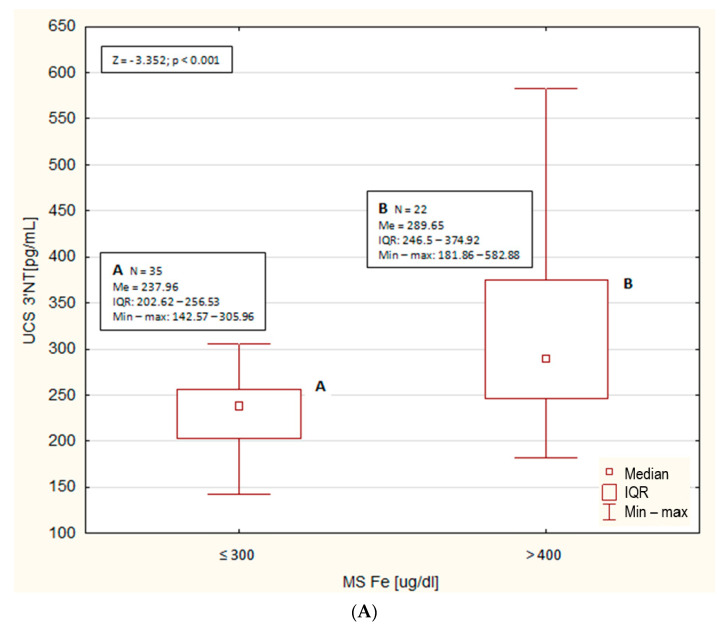

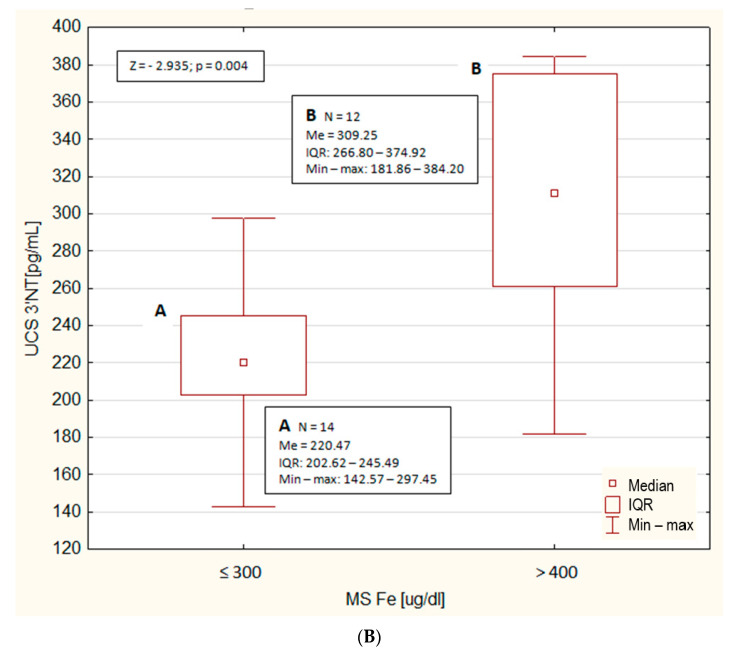
(**A**) Median concentrations (Me), interquartile range (IQR) and the range (Min-max) of 3′nitrotyrosine in cord blood serum (UCS 3′NT) of all newborns (regardless of sex) in relation to the concentration of Fe in the MS of ≤300 and >400 μg/dL. Descriptive statistics of subgroups (A and B), value of Mann–Whitney test (Z) and *p* value are presented in frames. (**B**) Median concentrations (Me), interquartile range (IQR) and the range (Min-max) of 3′nitrotyrosine in cord blood serum (UCS 3′NT) of male newborns in relation to the concentration of Fe in the MS of ≤300 and >400 μg/dL. Descriptive statistics of subgroups (A and B), value of Mann–Whitney test (Z) and *p* value are presented in frames.

The scatterplots of the concentration of 3′NT in the UCS in relation to the concentration of Fe in MS were analysed for all newborns (regardless of sex), female newborns and male newborns. On the scatterplots concerning all newborns and male newborns (in contrast to the scatterplot concerning female newborns), two clearly separated subgroups were observed with the cut-offs for the concentration of Fe in the MS of ≤300 and >400 μg/dL (no cases for MS Fe > 300 and ≤400 μg/dL). In the form of a boxplot, the median concentrations of 3′NT in the UCS in all newborns (regardless of sex) in relation to the concentration of Fe in the MS ≤ 300 and > 400 μg/dL and the results of Mann–Whithey test between subgroups are presented on the Figure 3A. A significantly lower concentration of 3′NT in the UCS (*p* < 0.001) was demonstrated in newborns whose mothers were characterized by serum Fe concentration up to 300 μg/dL compared to those of mothers with serum Fe concentration higher than 400 μg/dL. 

In the form of a boxplot, the median concentrations of 3′NT in the UCS of male newborns in relation to the concentration of Fe in the MS of ≤300 and >400 μg/dL and the results of Mann–Whitney test between subgroups are presented on the Figure 3B. A significantly lower concentration of 3′NT in the UCS (*p* = 0.004) was demonstrated in male newborns whose mothers were characterized by a serum Fe concentration up to 300 μg/dL compared to those of mothers with a serum Fe concentration higher than 400 μg/dL. 

## 4. Discussion

Results of this study showed a positive correlation between the level of OS in newborns and the concentration of Fe in MS. Moreover, a very high Fe concentration in the blood serum of pregnant women, exceeding 400 µg/dL, seemed to increase the level of OS in newborns. Importantly, a significant relationship was observed, especially in male neonates. 

Although, according to the author’s knowledge, there are no studies available in the literature strictly regarding the relationship between MS Fe concentration (or another marker of body Fe saturation) and the level of OS in newborns, some relationships regarding this issue have been clarified. 

Both Fe deficiency and excess during pregnancy are presented as unfavourable factors for foetal development, and the relationship is defined as U-shaped [64,65]. However, an excessive concentration of Fe acts as one of the key pro-oxidative factors by driving the Fenton reaction and the production of highly reactive hydroxyl radicals [13]. It has been shown that Fe supersaturation in the prenatal period may increase the risk of miscarriage, prematurity, low birth weight, and being small for gestational age (SGA) [64,66], as well as disturb the development of the foetal brain [67,68], and OS was demonstrated as one of the probable mechanisms leading to these abnormalities [64,69]. 

It is also known from a few reports that there is a relationship between the state of Fe saturation and the level of OS in pregnant women. Mannaerts et al. (2018) showed a positive correlation between the concentration of ferritin and superoxide anion in the blood of pregnant women [70]. In turn, Aly et al. (2016) found a positive correlation between the concentration of Fe and malondialdehyde, as well as the concentration of ferritin and total antioxidant capacity (TAC), in the blood of pregnant women [71]. These results prove the pro-oxidative properties of Fe, the increase in concentration which is associated with an increase in the production of free radicals and lipid peroxidation, and the mobilization of antioxidant mechanisms.

Much more research has been conducted on the effects of dietary Fe supplementation of pregnant women on the level of OS in their body. The results, however, are polarized, depending on the initial state of Fe saturation. In pregnant women not suffering from Fe deficiency anaemia (IDA), prophylactic Fe supplementation clearly induced OS [72,73] and limited the antioxidant capacity of the organism [73,74,75]. The pro-oxidative effect probably resulted from the oversaturation of the body with free Fe, the concentration of which exceeded the sequestration capacity of Fe-binding proteins, thus leading to the intensification of the Fenton and Haber–Weiss reactions and the production of ROS/RNS damaging biomolecules [76]. In turn, the use of dietary Fe supplementation in pregnant women with IDA had a beneficial antioxidant effect, with a significant decrease in the concentration of OS markers and an increase in the activity of antioxidant mechanisms [71,77]. Considering that Fe deficiency anaemia itself is a state of OS associated with an increased concentration of OS markers in the blood and a weakened activity of antioxidant mechanisms [71,78], equalization of Fe saturation to the optimal level, presented by pregnant women without IDA [71], seems to be a likely cause of lowering the level of OS after Fe supplementation in pregnant women with IDA. 

Negative effects of prophylactic Fe supplementation in pregnant women without IDA have also been observed in their newborn babies, who are significantly more often born with SGA [79] and with low birth weight (LBW) [80]. Moreover, birth weight and gestational age at birth were significantly lower in newborns of mothers supplementing Fe in higher doses compared to those who consumed less Fe [80]. 

Contrary to the current WHO recommendations regarding dietary Fe supplementation in pregnant women at a dose of 30–60 mg daily throughout pregnancy, regardless of the state of Fe saturation in the body [81], the results presented in the literature on the effects of Fe supplementation in pregnant women demonstrated that it should not be used in women without IDA [82]. It is emphasized that the health consequences of supplementation strictly depend on the initial state of Fe saturation in the bodies of pregnant women [64,83]. While supplementation is beneficial in women with anaemia, such an intervention may be detrimental in women who are well-nourished with Fe.

The results of studies carried out in various research centres have therefore shown that prenatal Fe supersaturation promotes OS in the bodies of pregnant women [70,71] and disturbs the normal growth and development of the foetus [64,66,67,68], which was also reflected in the results of studies on Fe supplementation in pregnant women without IDA [72,73,74,75,79,80]. The present study, in turn, demonstrated that the excessive concentration of Fe in the blood of pregnant women, exceeding 400 μg/dL, contributes to an increased level of OS in neonates, as well as a generally positive correlation between these variables. 

The results of this study also showed a negative correlation between the level of OS in male newborns and the concentration of Cu in MS. There are no reports in the available literature that would allow a direct comparison of the obtained results. 

It is known, however, that Cu, as a transition metal, can exhibit both antioxidant properties through the induction of Cu-dependent antioxidant enzymes (including Cu-Zn SOD, Se GPx and CAT) and pro-oxidative properties by participating in free radical reactions. Therefore, an insufficient Cu nutrition status results in the impairment of antioxidant mechanisms [50,52,53], while its excessive concentration promotes the production of ROS/RNS [48]. Hence, the relationship between the nutritional status of Cu and the level of OS is U-shaped. 

It has been established that both inadequate and excessive concentrations of Cu in the blood of pregnant women are associated with adverse health effects for the mother and the child. In pregnant women with pre-eclampsia, the pathogenesis of which is clearly associated with an elevated level of OS [84], an increased [85] or reduced [86,87,88] Cu concentration in maternal blood was observed. A lower blood Cu concentration was also found by Biswas et al. (2016) in women with gestational hypertension compared to that in healthy pregnant women [89]. Neural tube defects, including foetal anencephaly, also appear to be associated with both maternal Cu deficiency [90,91] and its excess [92], and OS was suggested as one of the probable mechanisms [55,93,94]. Moreover, Wilson et al. (2018) showed that pregnant women with higher blood Cu levels (3rd tertile) had a significantly higher risk of gestational complications (pre-eclampsia, gestational diabetes, premature birth and/or SGA) compared to pregnant women whose Cu blood concentration was within the 1st and 2nd tertiles. The authors also found a positive correlation between the concentration of Cu and acute phase proteins (APPs) in the blood of pregnant women, and they considered that to be evidence of the pro-inflammatory and pro-oxidative mechanisms of the development of these complications [95]. The case study presented by Walker et al. (2011) suggests that excessive Cu concentration in the blood of pregnant women can lead to autism and cardiological problems in their offspring in childhood [96]. Similar problems are diagnosed in Wilson’s disease associated with disturbed metabolism of Cu leading to the supersaturation of the body with this mineral element [97]. However, in the study by Walker et al. (2011) [96], there is no mention of a potential mechanism linking an excessive concentration of Cu in the blood of pregnant women to autism and cardiological problems in the offspring, aetiopathogenesis of cardiovascular dysfunctions as well as autism spectrum disorders (ASD) [6,98]. Moreover, Shen et al. (2015) observed a decreased Cu concentration in the blood of pregnant women whose children were born with intrauterine growth restriction (IUGR) [99]. Additionally, Pathak and Kapil (2004) summarized the literature, recognizing the deficiency of trace elements, including Cu, as one of the causes of infertility, miscarriages, gestational hypertension, placental abruption, premature rupture of membranes (PROM), congenital malformations and low birth weight (LBW) [100]. Li et al. (2018) also showed that both newborns with low (1st tertile) and high (3rd tertile) concentrations of Cu in the UCS had a significantly increased risk of being born prematurely or late, respectively [101]. Other researchers demonstrated a similar positive correlation between the concentration of Cu in the umbilical cord blood and foetal age [102,103,104]. 

The results presented in the discussion confirm the U-shaped relationship between the concentration of Cu in the blood of pregnant women and the health effects related to OS, representing the left [86,87,88,89,90,91,99,100,101] or the right [85,92,95,96,101] arm of the curve. Taking into account that the results obtained in the present study concerned a group of women characterized mostly by insufficient Cu concentrations (66.7%), with a median value of 78.02 (IQR—44.00–123.20) μg/dL, the demonstrated correlation seems to support the U-shaped nature of the curve of the discussed relationship, representing its left arm, and suggesting the concentration of 3′NT in umbilical cord blood may decrease as the concentration of Cu in maternal blood approached the optimal values.

In the author’s own research, the nutritional status of pregnant women seems to modify the level of OS mainly in male newborns. It would confirm the conclusions presented by Lavoie and Tremblay (2018) after reviewing studies from 1990 to 2017 on the relationship between the level of OS and the sex of newborns. They found that the blood concentrations of OS markers were higher and the antioxidant capacity was lower in male neonates compared to those in female neonates [105]. In animal models, it was also unequivocally demonstrated that the susceptibility of the foetus to OS was sex-dependent. Increased antioxidant activity in response to placental insufficiency [106], prenatal betamethasone exposure [107] and malnutrition [108] were observed only in female neonates. This proves more efficient compensation mechanisms in the female sex and greater sensitivity to unfavourable conditions of the intrauterine environment and OS in the male sex. Potentially protective effects may include female sex hormones (oestrogens) [109,110,111], as well as a higher adaptive capacity of the female placenta [112], and more adaptable strategies of the development of female foetuses [113] to the stressful conditions of the intrauterine environment. 

It is worth noting that the present study, according to our best knowledge, examined for the first time the OS in newborns in relation to the status of mineral nutrition in pregnancy. Our study, however, has several limitations, including moderately large study group (below minimum sample size [114,115]), a cross-sectional nature instead of a multiple model, and no adjustment for potential confounding factors (e.g., obesity, older maternal age), therefore further extensive studies on this issue are needed. 

## 5. Conclusions

In conclusion, a significant relationship between the nutritional status with mineral elements in pregnant women and the level of OS in their newborn children has been demonstrated. A significantly excessive concentration of Fe in the blood serum of pregnant women, exceeding 400 µg/dL, seemed to promote OS in newborns. Moreover, results of the study seem to support a U-shaped relationship between the level of neonatal OS and the mother’s blood Cu concentration, suggesting the concentration of 3′NT in umbilical cord blood may decrease as the concentration of Cu in maternal blood approached the optimal values. Interestingly, the impact of the nutritional status of pregnant women on the level of OS was observed mainly in male newborns. 

The results of the study showed the need to develop strategies to optimize the nutritional status of pregnant women. Implementation of these strategies could contribute to reducing the risk of pre- and neonatal OS and its adverse health effects in the offspring, including disorders classified as “oxygen radical disease in neonatology”, as well as cardiometabolic and immune diseases in later life resulting from foetal programming.

However, the presented conclusions must be confirmed in further studies. It would be greatly recommended to conduct them longitudinally throughout pregnancy, on a larger population of pregnant women and their newborn children, exceeding the minimum sample size. Moreover, in further studies, more markers of OS should be included, e.g., the concentrations of the products of protein, lipid and DNA oxidation, the levels of antioxidants, the activity of antioxidant enzymes, and various oxidant and antioxidant indicators. Additionally, as there are many potential confounding factors that may influence OS in newborns, such as maternal age, diet, physical activity, maternal BMI, gestational weight gain and others mentioned in the introduction [7,8,9,10], it would be meaningful to include them into a fully adjusted multivariate model. 

Further extensive research on this issue, conducted on a sufficiently large study group and adjusted for potential confounding factors will guarantee the scientific credibility of the results, enable the generalization of the results from the sample to the entire population and enable the application of the results to create nutritional recommendations for pregnant women aimed at reducing the levels of OS in newborns. The results obtained in this study should be considered as an important turn signal for future research. 

## Figures and Tables

**Table 1:** Characteristics of mothers and newborns (N = 57).

	Mean	SD	Median	IQR	Range
**Maternal characteristics**					
Maternal age [years]	33.01	4.90	33.00	30.00–35.00	19.00–50.00
Pre-pregnancy BMI [kg/m^2^]	23.21	4.24	22.09	20.19–25.24	16.73–40.12
Gestational weight gain [kg]	14.88	4.62	14.00	12.00–18.00	5.00–25.50
MS Mg [mg/dL]	1.24	0.14	1.22	1.15–1.33	0.93–1.66
MS Ca [mg/dL]	17.33	3.21	17.61	15.93–18.77	8.34–26.94
MS Fe [µg/dL]	382.0	264.7	328.5	169.0–555.0	0.01–1603.0
MS Cu [µg/dL]	83.31	56.0	78.02	44.00–123.2	0.00–248.1
MS Zn [µg/dL]	19.36	16.16	17.65	0.00–32.80	0.00–56.30
**Neonatal characteristics**					
Gestational age [weeks]	38.78	0.88	39.00	38.00–39.00	37.00–41.00
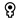	38.89	0.96	39.00	38.00–39.00	37.00–41.00
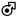	38.65	0.78	39.00	38.00–39.00	37.00–41.00
Birth weight [g]	3466.9	447.7	3405.0	3150.0–3710.0	2490.0–4980.0
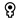	3446.3	424.4	3380.0	3140.0–3680.0	2580.0–4980.0
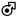	3490.0	476.6	3460.0	3170.0–3730.0	2490.0–4660.0
UCS 3′NT [pg/mL]	255.7	74.13	244.1	214.6–272.4	142.6–582.9
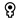	256.9	81.67	245.7	215.2–261.8	154.0–582.9
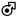	254.1	64.33	236.8	210.33–297.5	142.5 –384.2

MS—maternal serum, UCS—umbilical cord serum, 3′NT—3′nitrotyrosine, 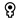 N = 31, 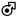 N = 26, IQR—interquartile range.

**Table 2:** The frequency of insufficient, optimal and excessive concentrations of selected mineral elements in the blood serum of pregnant women in relation to the reference values for pregnant women in the 3rd trimester *.

Mineral Elements	Reference Values *	Insufficient [%] ^1^	Optimal [%] ^2^	Excessive [%] ^3^
Mg [mg/dL]	1.1–2.2	15.8	84.2	-
Ca [mg/dL]	8.2–9.7	-	-	100
Zn [µg/dL]	50–77	96.5	3.5	-
Fe [µg/dL]	30–193	1.8	38.6	59.6
Cu [µg/dL]	130–240	66.7	31.5	1.8

* Ref. [58], ^1^ Insufficient—below the lower limit of the reference values, ^2^ Optimal—within the reference values, ^3^ Excessive—above the upper limit of the reference values.

**Table 3:** Spearman’s correlation between the concentration of selected mineral elements in maternal blood serum and the concentration of 3′nitrotyrosine in cord blood serum of all newborns, female newborns and male newborns.

Examined Mineral Elements	UCS 3′NT (pg/mL) (N = 57)		UCS 3′NT (pg/mL) 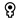 (N = 31)		UCS 3′NT (pg/mL) 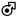 (N = 26)	
	rho	*p*	rho	*p*	rho	*p*
MS Mg (mg/dL)	0.045	0.740	−0.183	0.325	0.252	0.214
MS Ca (mg/dL)	0.108	0.423	0.036	0.848	0.123	0.550
MS Zn (µg/dL)	−0.042	0.768	−0.343	0.074	0.141	0.500
MS Fe (µg/dL)	0.233	0.093	−0.054	0.786	0.392	0.053
MS Cu (µg/dL)	−0.179	0.184	0.246	0.182	−0.509	0.008 *

MS—maternal serum, UCS—umbilical cord serum, Mg—magnesium, Ca—calcium, Zn—zinc, Fe—iron, Cu—copper, 3′NT—3′nitrotyrosine, 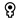
—female newborns, 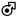
—male newborns, * statistically significant (*p* ≤ 0.05), _—at the edge of statistical significance.

**Table 4:** The concentration of 3′nitrotyrosine in umbilical cord serum in relation to the concentration of selected mineral elements in maternal serum (insufficient, optimal and excessive).

Examined Mineral Elements	UCS 3′NT [pg/mL]									MW-Z	*p*
	Insufficient			Optimal			Excessive				
	N	Me	IQR	N	Me	IQR	N	Me	IQR		
MS Mg [mg/dL]	9	244.05	227.23–261.70	48	245.06	214.88–275.00	0	---	---	−0.077	0.939
MS Ca [mg/dL]	0	---	---	0	---	---	57	245.49	215.21–272.42	---	---
MS Fe [µg/dL]	1	---	---	22	244.86	216.07–261.70	34	246.00	213.97–304.90	−0.741	0.459
MS Zn [µg/dL]	55	245.37	213.97–269.76	2	---	---	0	---	---	---	---
MS Cu [µg/dL]	38	239.87	214.55–274.40	18	249.34	218.63–266.20	1	---	---	−0.202	0.841

MS—maternal serum, UCS—umbilical cord serum, Mg—magnesium, Ca—calcium, Zn—zinc, Fe—iron, Cu—copper, 3′NT—3′nitrotyrosine, Me—median, IQR—interquartile range, MW-Z—result of Mann–Whitney test.

**Table 5:** The concentration of 3′nitrotyrosine in umbilical cord serum in relation to the concentration of calcium and zinc in maternal serum divided into quartiles.

Examined Mineral Elements	UCS 3′NT [pg/mL]												KW-H	*p*
	Q_1_			Q_2_			Q_3_			Q_4_				
	N	Me	IQR	N	Me	IQR	N	Me	IQR	N	Me	IQR		
MS Ca [mg/dL]	15	234.04	215.21–258.73	13	253.90	237.96–272.42	13	243.85	202.62–261.70	16	247.33	221.49–310.09	1.610	0.657
MS Zn [µg/dL]	14	252.01	232.01–310.09	16	229.28	202.62–272.42	13	245.49	210.33–266.20	14	249.81	237.96–310.56	1.735	0.629

MS—maternal serum, UCS—umbilical cord serum, Ca—calcium, Zn—zinc, 3′NT—3′nitrotyrosine, Me—median, IQR- interquartile range, KW-H—result of Kruskal–Wallis test.

## Data Availability

Data are available on reasonable request from the corresponding author.
